# Integrative systems and functional analyses reveal a role of dopaminergic signaling in myelin pathogenesis

**DOI:** 10.1186/s12967-020-02276-1

**Published:** 2020-03-02

**Authors:** Sujun Ding, Yun Gu, Yunyun Cai, Meijuan Cai, Tuo Yang, Shuangxi Bao, Weixing Shen, Xuejun Ni, Gang Chen, Lingyan Xing

**Affiliations:** 1 School of Medicine, Nantong University, Nantong, China; 2 Department of Ultrasound, Affiliated Hospital of Nantong University, Nantong, China; 3 Key Laboratory of Neuroregeneration of Jiangsu and the Ministry of Education, Co-innovation Center of Neuroregeneration, Nantong University, Nantong, China; 4 Department of Physiology, School of medicine, Nantong University, Nantong, China; 5 Department of Clinical Laboratory, Qilu Hospital of Shandong university, Shandong, China; 6 Department of Hand Surgery, China-Japan Union Hospital of Jilin University, Changchun, China; 7 Department of Physiology, School of medicine, Co-innovation Center of Neuroregeneration, Nantong University, Nantong, China; 8 Department of Anesthesiology, Affiliated Hospital of Nantong University, Nantong, China

**Keywords:** Dopaminergic signaling, myelin pathogenesis, genetics, transcriptome, zebrafish, *in vivo* analysis

## Abstract

**Background:**

Myelin sheaths surrounding axons are critical for electrical signal transmission in the central nervous system (CNS). Diseases with myelin defects such as multiple sclerosis (MS) are devastating neurological conditions for which few effective treatments are available. Dysfunction of the dopaminergic system has been observed in multiple neurological disorders. Its role in myelin pathogenesis, however, is unclear.

**Methods:**

This work used a combination of literature curation, bioinformatics, pharmacological and genetic manipulation, as well as confocal imaging techniques. Literature search was used to establish a complete set of genes which is associated with MS in humans. Bioinformatics analyses include pathway enrichment and crosstalk analyses with human genetic association studies as well as gene set enrichment and causal relationship analyses with transcriptome data. Pharmacological and CRISPR/Cas9 (clustered regularly interspaced short palindromic repeats/CRISPR-associated protein 9) genetic manipulation were applied to inhibit the dopaminergic signaling in zebrafish. Imaging techniques were used to visualize myelin formation *in vivo*.

**Results:**

Systematic analysis of human genetic association studies revealed that the dopaminergic synapse signaling pathway is enriched in candidate gene sets. Transcriptome analysis confirmed that expression of multiple dopaminergic gene sets was significantly altered in patients with MS. Pathway crosstalk analysis and gene set causal relationship analysis reveal that the dopaminergic synapse signaling pathway interacts with or is associated with other critical pathways involved in MS. We also found that disruption of the dopaminergic system leads to myelin deficiency in zebrafish.

**Conclusions:**

Dopaminergic signaling may be involved in myelin pathogenesis. This study may offer a novel molecular mechanism of demyelination in the nervous system.

## Background

Myelin sheaths surrounding axons ensure rapid action potential conduction. Myelin pathogenesis derived from developmental deficits or demyelination is deleterious in the central nervous system (CNS), leading to irreversible and progressive neurological decline. Myelin deficits have been found in multiple neurological disorders, such as Parkinson’s disease (PD), schizophrenia, and multiple sclerosis (MS) [1, 2], many of which have disturbed dopaminergic system. However, the relationships between dopaminergic signaling and myelin defects are not well understood.

PD and schizophrenia are neurological disorders at least partially attributed to disrupted dopaminergic signaling [3, 4]. In patients with PD, abnormal CNS connectivity was observed in several brain regions [5, 6]. White matter defects indicate that myelin abnormalities underlie some aspects of PD. The potential roles of dopaminergic signaling in white matter and myelin integrity were further elucidated in patients with schizophrenia. The antipsychotic drug clozapine has been shown to improve white matter integrity in schizophrenia by way of blocking serotonin and dopamine receptors [7]; Another imaging study specifically links dopamine D2/D3 receptor density to myelin indices in normal and schizophrenic white matter [8]. These indicate a potential role of dopaminergic signaling in myelin deficits.

MS is a neurological disorder characterized by demyelination and axon loss. Interestingly, concomitance of MS and PD as well as MS and schizophrenia has been observed [9, 10]. Altered dopamine receptor levels were found in the blood of patients with MS [11, 12]. Moreover, modulation of dopamine receptors can promote or prevent experimental autoimmune encephalomyelitis (EAE) in mouse, a rodent model for human MS [13, 14]. Based on these, we hypothesize that dopaminergic signaling may be important for demyelination in MS.

In this study, we described a systems analysis which integrate multi-source-based data from human genetic association studies and transcriptome data in patients with MS to investigate the role of dopaminergic signaling in MS. This unbiased approach combined with *in vivo* functional analysis will provide us with insight into a role of dopaminergic signaling in MS.

## Materials and methods

### Identification of MS-related genes

MS-related genes were obtained by a systematic analysis of the human genetic association studies deposited in Pubmed (https://www.ncbi.nlm.nih.gov/pubmed) [15]. Similar to references [16–18], we queried for publications about MS with the terms (Multiple sclerosis [MeSH]) and (polymorphism [MeSH] or genotype [MeSH] or alleles [MeSH]) not (neoplasms [MeSH]). 2428 publications in total were found by June 8^th^, 2018. We selected only those genes reported to be associated with MS by manually reviewing abstracts and the full reports if the abstract was not clear. When multiple genes reported to act together were associated with MS, all of these genes were included. In addition, genes with a genome-wide significance level from the genome-wide association study (GWAS) were also included.

### Enriched pathway and aggregated category analysis

Enriched Kyoto Encyclopedia of Genes and Genomes (KEGG) pathways of MS gene sets were generated based on the latest KEGG database 90.1 (https://www.genome.jp/kegg/pathway.html) [19]. Hypergeometric distribution was used to determine enrichment of the specified gene sets in pathways. P values were corrected with the Benjamini-Hochberg procedure. Only those pathways with >=10 target genes were examined further.

### Gene interaction analysis

Gene interactions between genes in the dopaminergic synapse pathway (DS) and other literature-curated (oLC) genes in MS were analyzed with datasets from Pathway Commons (http://www.pathwaycommons.org/) [20, 21] and high-confidence human interactome [22]. In Pathway Commons (http://www.pathwaycommons.org/) [21], only four common types of interactions were considered: controls-state-change-of, controls-expression-of, controls-transport-of, and controls-phosphorylation-of. In Pathway Commons (http://www.pathwaycommons.org/) [21], directed interactions are provided; that is, interactions between genes of DS and oLC were analyzed with DS either as upstream or downstream genes. In high-confidence human interactome, undirected interactions are provided.

### Transcriptome analysis

To study the transcriptional profile of brain lesions in patients with multiple sclerosis, we downloaded microarray data in the GEO database (https://www.ncbi.nlm.nih.gov/geo/) [23] from GSE26927 for grey matter lesions and from GSE38010 for white matter lesions. GSE26927 is the microarray data on the Illumina human Ref-8 v2.0 expression beadchip [24, 25]. Data was normalized by Quantile algorithm. 15373 probes were detected after filtering low expression with a detection p value <0.01. GSE38010 is data from the Affymetrix Human Genome U133 Plus 2.0 Array [26]. Data was normalized by the Rosetta error models. Only probes with log2 transformed normalized signal >=6 in at least one sample were considered, of which 31275 probes were detected.

### Gene set enrichment analysis

Differentially expressed gene sets in GSE26927 and GSE38010 were identified with the ROAST test (ROAST: rotation gene set tests for complex microarray experiments) [27]. We used the ‘mixed’ test, in which the directionality of changes in expression was not considered. Multiple gene sets were selected based on their importance in regulation of dopaminergic signaling from Gene Ontology: the dopamine catabolic process (GO:0042420), dopamine receptor activity (GO:0004952), dopamine receptor binding (GO:0050780), dopamine secretion (GO:0014046), dopamine transport (GO:0015872), dopamine uptake (GO:0090494), L-dopa decarboxylase activity (GO:0036468), regulation of the dopamine biosynthetic process (GO:1903179), and regulation of the dopamine receptor signaling pathway (GO:0060159).

### Gene set causal relationship analysis

To calculate the relationship between two gene sets, “Super Gene Set” causal relationship analysis was performed as previously described [28]. Gene expression values were discretized as follows: the highest 1/3 absolute gene expression was converted to +1, the lowest 1/3 absolute expression values were represented as –1, while the other 1/3 in between were assigned to 0. Gene set-gene set correlation was determined by hypergeometric distribution, in which p values <0.05 were considered significantly different.

### Dopamine enzyme linked immunosorbent assay (ELISA) on zebrafish larvae

Briefly, 25 larvae were collected for each sample, and both control and experimental groups were performed in triplicate. Tissues were minced, 200 ul PBS was added, and then they were sonicated by an ultrasonic cell disrupter. The homogenates were then centrifuged and the supernatant was collected. Dopamine levels were determined with the Dopamine (DA) ELISA kit (Biovision) following the manufacturer’s instructions. Dopamine values were normalized to larvae weight, and were expressed as ng dopamine/larvae weight.

### *Otpa/otpb* CRISPR/Cas9

One-cell stage embryos from *Tg(mbp:egfp-caax)* [29] outcrossed to wild-type were injected with 300 pg of Cas9 mRNA and 100 pg of *otpa* and *otpb* sgRNA. sgRNA was targeted to the following sequences in *otpa* and *otpb*: GGCGGCCGCAGCAGCCAT and GGCCGCGGCTGGGATGCCGG, respectively. pCS2 with full length Cas9 was linearized by XbaI [30]. Capped Cas9 mRNA was synthesized using the mMESSAGE mMACHINE mRNA transcription synthesis kit (Ambion) and purified with the Megaclear kit (Thermo Fisher Scientific). The pMD 19-T vector with a gRNA scaffold was used for sgRNA synthesis. Double-stranded DNA for specific gRNA synthesis was PCR amplified as follows: *otpa* sgRNA forward: GGGGCGGCCGCAGCAGCCAT, *otpb* sgRNA forward: GGCCGCGGCTGGGATGCC GG, and universal reverse: AAAAAAAGCACCGACTCGGTGCCAC. Amplicons were purified with the DNA clean & concentrator kit (Zymo Research). After purification, sgRNA was synthesized using the Megashortscript^TM^ kit (Thermo Fisher Scientific) and purified with the Megaclear kit (Thermo Fisher Scientific).

### *Otpa/otpb* mutagenesis verification

Genomic DNA from 24 hpf embryos was extracted and tested as previously described [31]. Briefly, embryos were lysed with 50 mm NaOH at 95°C for 25 min to obtain PCR templates. DNA surrounding the *otpa* and *otpb* sgRNA regions was amplified and sequenced. To confirm the frequency of mutagenesis, the targeted DNA regions in F0 founders were cloned into pCR4-TOPO TA (ThermoFisher). Plasmids were isolated from individual colonies and Sanger sequencing was performed (Genewiz, Inc).

### Microscopy and image analysis

Images were acquired and analyzed as previously described [32]. Live *Tg(mbp:egfp)* embryos were immobilized in 0.8% low-melt agarose and mounted on a petri dish. A confocal z-stack was taken in the regions of interest using exactly the same confocal settings (20×water-immersion objective, same PMT and imaging speeds). Confocal stacks of maximum intensity were projected in ImageJ. For quantification of myelination, ten slices of z-stacked images were used with the same lower and upper thresholds set to define parts with/without myelin formation. The myelin in the spinal cords was measured along the image window.

## Results

### Systematic analysis of genetic association studies in multiple sclerosis (MS)

**Fig. 1:**
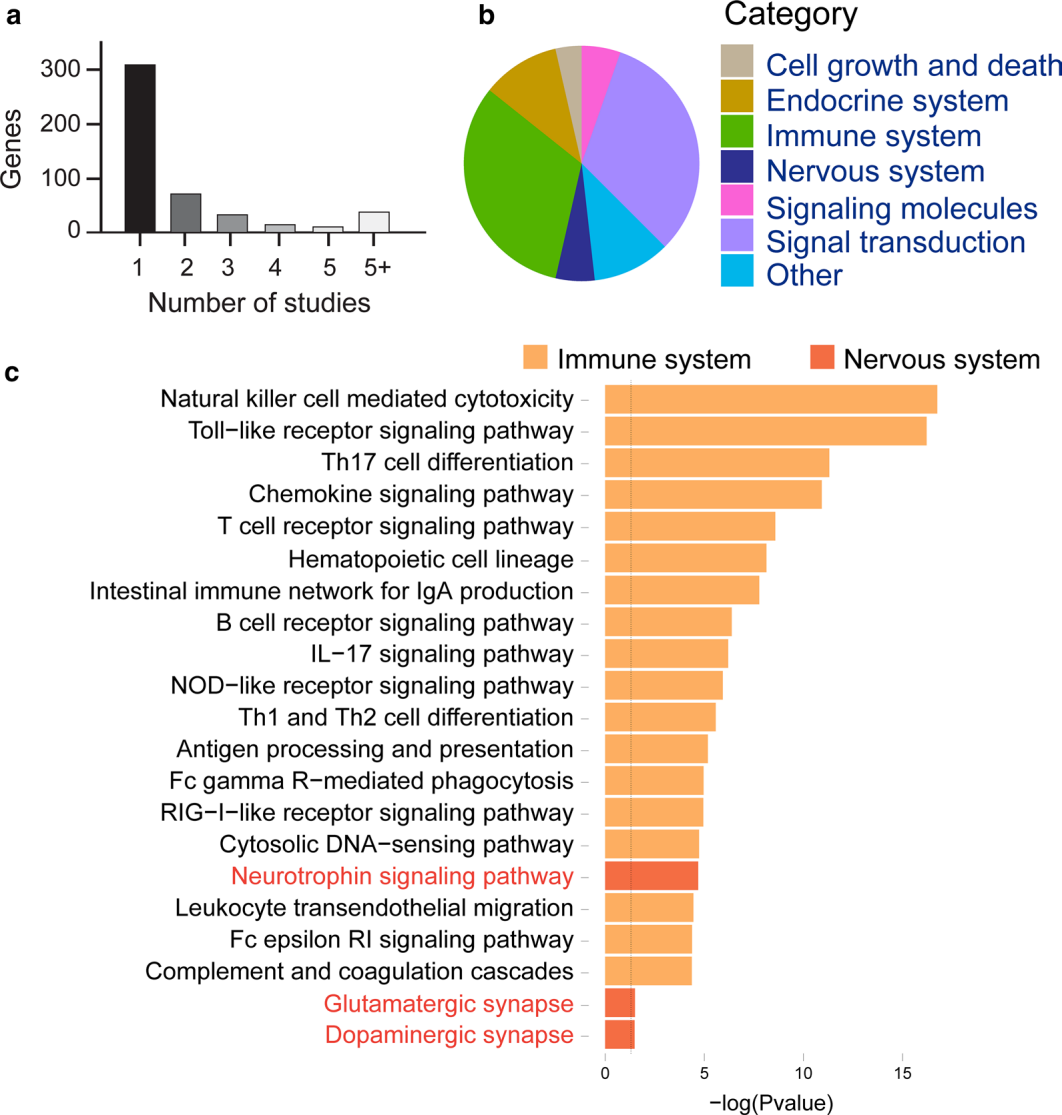
Functional enrichment analysis of literature-curated (LC) genes in multiple sclerosis (MS). **a** Number of studies about genes associated with multiple sclerosis (MS) in literature-curated (LC) genes. **b** Main categories aggregated by the enriched KEGG pathway. **c** Pathways of the immune system and nervous system are enriched in the LC gene set

In order to establish a complete list of genes associated with MS, we conducted a systematic analysis of human genetic association studies, examining 2428 papers published through June 2018. In 1254 publications, 469 genes had a significant association with MS (Additional file 1). Of these genes, 164 genes were associated with MS in more than one study (Figure 1a). Among them were the HLA-DRB1 gene and the T-cell receptor gene (TCR), which are important in the pathogenesis of MS [33–35]. The diversity of MS-related genes indicates the complexity and heterogenity of this disease.

### Functional enrichment analysis

To better understand the molecular mechanisms in MS, we performed functional enrichment analysis of MS-related genes. We found 56 enriched KEGG pathways in the candidate genes (Additional file 2). These pathways included but were not limited to those related to the immune response (e.g., natural killer cell mediated cytotoxicity, Toll-like receptor signaling pathway, Th17 cell differentiation), signaling molecules and interaction (e.g., cytokine-cytokine receptor interaction, cell adhesion molecules), and the nervous system (e.g., the neurotrophin signaling pathway and the dopaminergic synapse signaling pathway) (Figure 1b, c; Additional file 2). These results indicated complicated interactions among multiple systems in MS, which were consistent with what has been found previously [36].

Great efforts have been made to study the role of the immune response in MS, which was considered to be an inflammatory-mediated disease. However, we are particularly interested in the signaling pathways in the nervous system, because new evidence suggests that MS is primarily a neurodegenerative disease [37, 38]. As expected, in the nervous system category, the neurotrophin signaling pathway, disruption of which is known to account for neural degeneration [39, 40], was enriched (Figure 1c). The glutamatergic synapse pathway is also enriched, which is consistent with the findings that glutamate is elevated in MS and that its receptor antagonist reduced secondary damage in EAE [37, 41–43]. Finally, the dopaminergic synapse signaling pathway, known for its role in multiple neurological disorders [3, 4], is enriched (Figure 1c), leading us to hypothesize that dopaminergic signaling may be important in MS.

### Validation of dopaminergic gene sets in MS

**Fig. 2:**
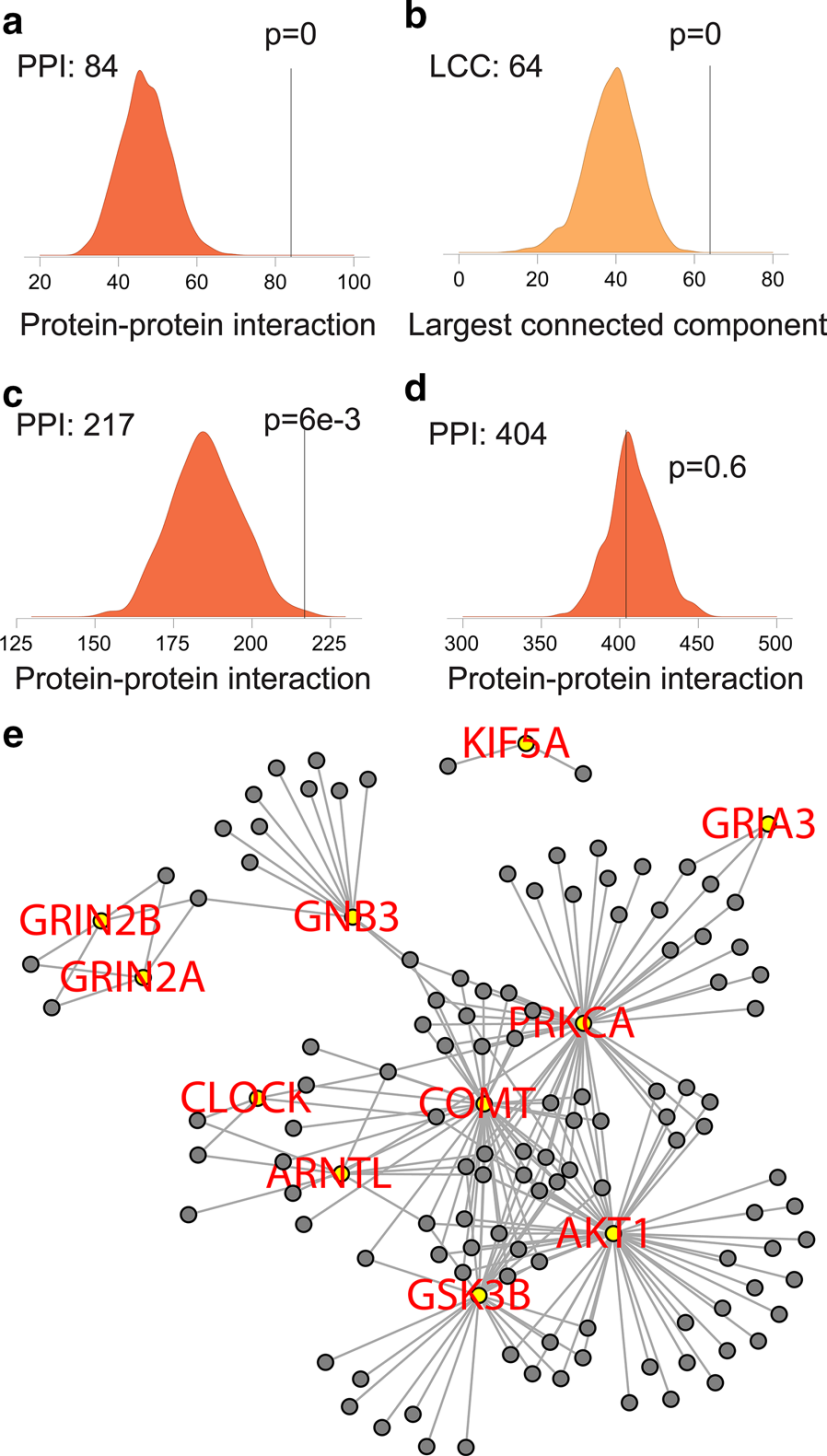
Networks between DS and other literature-curated (oLC) genes in MS. **a**, **b** in the high-confidence human interactome database. **a** Number of protein–protein interactions (PPIs) between DS and oLS (Line) and 1000 randomized networks, revealing high levels of interaction between DS and oLS. **b** Size of the largest connected component (LCC) between DS and oLS (Line) and 1000 randomized networks, revealing a larger subnetwork between DS and oLS. **c**, **d** In the Pathway Commons database. Number of protein–protein interactions (PPIs) between DS and oLS (Line) and 1000 randomized networks with the DS gene set as upstream (**c**) or downstream (**d**). **e** Gene interaction between DS and oLC genes in MS. Yellow nodes-DS genes; Grey nodes-oLC genes. Edges represent their interaction

To validate the biological significance of the dopaminergic synapse (DS) gene set, we first evaluated the interactions between the protein products of DS genes and other literature-cured (oLC) MS genes with datasets either from Pathway Commons (http://www.pathwaycommons.org/) [21] or high-confidence human interactome. Datasets from high-confidence human interactome offers undirected protein-protein interactions, while datasets from Pathway Commons (http://www.pathwaycommons.org/) [21] provides directed protein-protein interactions [20][22]. Protein-protein interactions between DS and oLC were significantly enriched relative to random expectations when analyzed with the datasets from high-confidence human interactome (Figure 2a; Additional file 3). Interestingly, when we tested the protein interactions between DS and oLC with the directed datasets from Pathway Commons (http://www.pathwaycommons.org/) [21], significant enrichment was observed when DS genes were used as upstream but not downstream bait (Figure 2c, d; Additional file 4), indicating a potentially causative role of dopamine in MS pathogenesis. Next, we calculated the size of the largest connected component (LCC), which represents the density of interconnections, and found that the DS forms a significantly larger network with oLC genes relative to random expectations (Figure 2b). High levels of interaction between DS and oLC genes (Figure 2e) indicate an important role of the DS pathway in MS.

To identify pathways directly interacting with the DS pathway, PathNet (the Pathways based on Network information) was applied to calculate their inter- and intrapathway relationships [44]. We found that the dopaminergic synapse pathway has significant interaction with multiple pathways enriched in multiple sclerosis, including the complement and coagulation cascade, Rap1 signaling, and neuroactive ligand receptor interaction (Additional file 5).

### Transcriptome analysis of dopaminergic gene sets

**Fig. 3:**
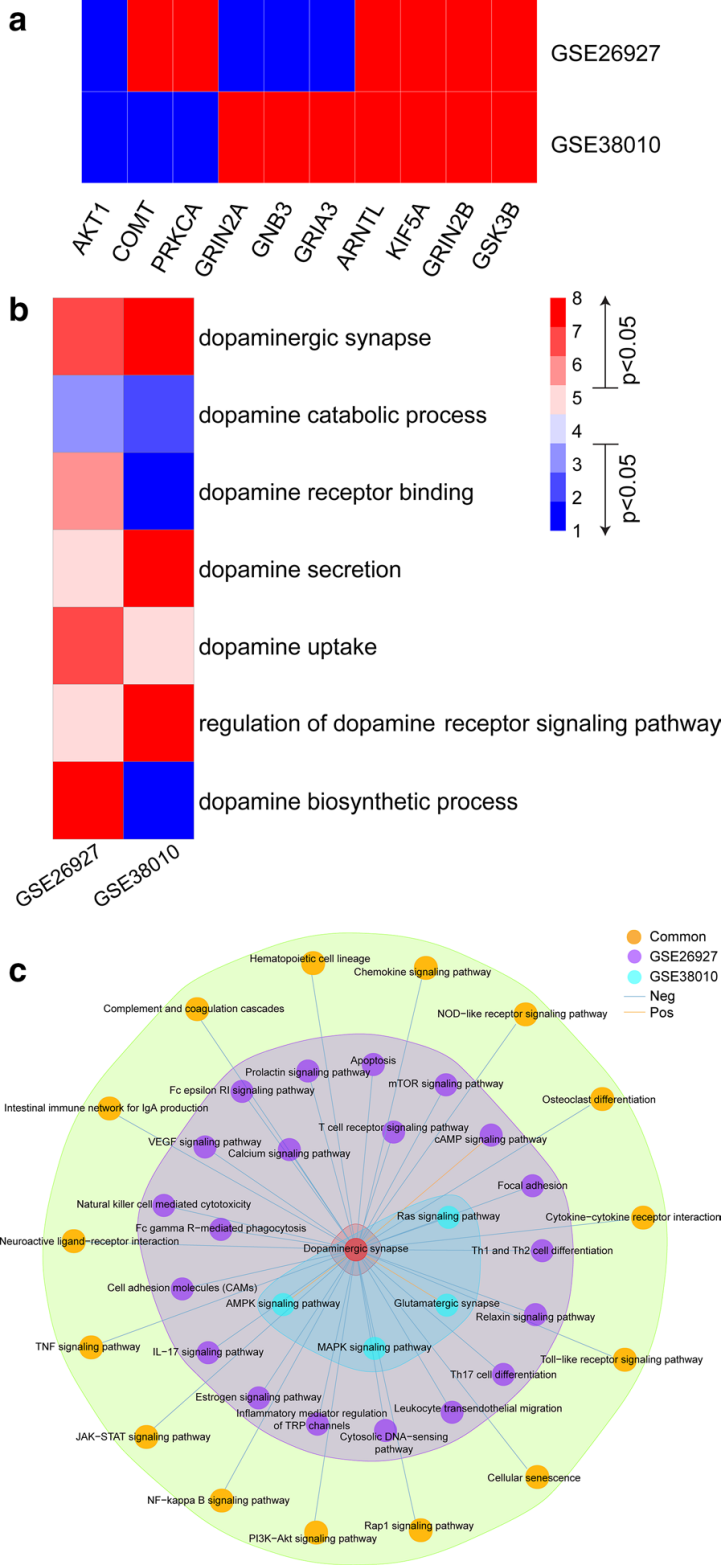
Transcriptome analysis of the DS gene set. **a** Expression of genes in the DS pathway in the transcription datasets GSE26927 and GSE38010. Red corresponds to upregulated expression; blue corresponds to downregulated expression. **b** Expression changes of multiple dopaminergic gene sets. Red represents upregulation; blue represents downregulation. Numbers 1–3 and 6–8 indicate p values < 0.05. **c** Pathway associations between DS and oLC in two transcriptome databases

Though MS was traditionally considered to be a white matter disease, some clinical symptoms are attributed to grey matter lesions [45, 46]. We hypothesized that if dopaminergic signaling was critical in MS, the expression of related gene sets would be altered in patients with MS. To test that, we retrieved microarray datasets GSE26927 for grey matter and GSE38010 for white matter in MS patients [24–26]. Expression changes of multiple genes in dopamine pathways were observed in either white matter or grey matter lesions (Fig. 3a). To comprehensively understand the changes of dopaminergic signaling, we used the ROAST test [27] to measure several gene sets critical for dopaminergic signaling: the dopamine catabolic process (GO:0042420), dopamine receptor activity (GO:0004952), dopamine receptor binding (GO:0050780), dopamine secretion (GO:0014046), dopamine transport (GO:0015872), dopamine uptake (GO:0090494), L-dopa decarboxylase activity (GO:0036468), regulation of the dopamine biosynthetic process (GO:1903179), regulation of the dopamine receptor signaling pathway (GO:0060159), as well as the dopaminergic synapse pathway. Interestingly, the expression levels of most dopamine gene sets show discrepancies between white matter and grey matter lesions. The dopamine uptake gene set was upregulated in grey matter (p=0.015), but did not show differential expression in white matter. Expression of the dopamine catabolic process and dopamine receptor binding were significantly downregulated (p=0.044, 0.001, respectively) and dopamine secretion and regulation of the dopamine receptor were upregulated (p=0.001, 0.006, respectively) in white matter, but were not altered in grey matter. The expression changes of the biosynthetic process gene set were opposite in grey and white matter (p=0.004, 0.005, respectively). The dopaminergic synapse gene set was the only one significantly upregulated in both grey and white matter (p=0.01, 0.037, respectively) (Fig. 3b).

Next, we tested the association between the DS pathway and other pathways enriched in MS based on the expression data. This reveals a regulatory relationship regardless of direct or indirect interactions between pathways [28]. We found quite a few pathways correlated with the DS pathway in either grey or white matter (Fig. 3c, Additional file 6). The neuroactive ligand-receptor, Rap1 signaling pathway, and complement and coagulation cascades were all negatively associated with the DS pathway in these two datasets. Of note, in white matter grey lesions, more pathways were associated with the DS pathway in MS, including the cyclic adenosine monophosphate (cAMP) signaling pathway, cell adhesion molecules, Fc gamma R-mediated phagocytosis, and Th17 cell differentiation. These indicate that MS pathology may exhibit distinct mechanisms in grey and white matters (Fig. 3c).

### In vivo functional analysis

Demyelination can be partially recapitulated by defects in myelination during development. Multiple studies have used developmental models to study the mechanisms of the pathology of myelination [47, 48]. To confirm a role of dopaminergic signaling in myelination, we used the zebrafish transgenic line *Tg(mbp:egfp)* to label myelin. In this line, enhanced green fluorescent protein (*eGFP*) is expressed under the control of the *mbp* (myelin basic protein) promoter, which has been widely used for assessing myelin development and dysmyelination [29, 48–50]. In *Tg(mbp:egfp)*, myelin can be specifically expressed along the spinal cord, where dopaminergic innervation is observable [51].

**Fig. 4:**
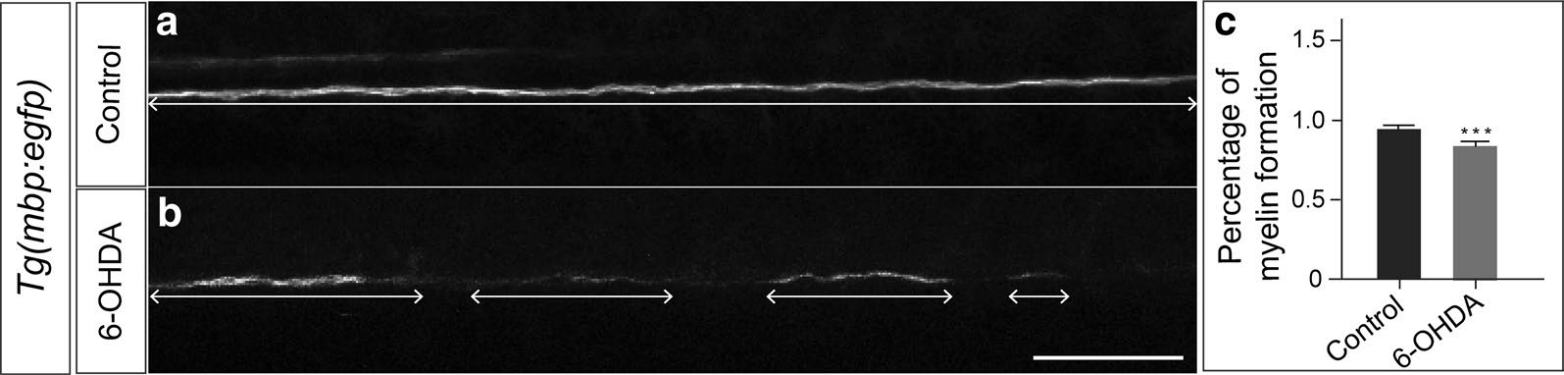
6-OHDA disrupts myelin. **a**, **b***Tg* (*mbp:egfp*) at 72hpf by confocal microscopy imaging, maximum intensity z-stack projections. **a** Control. **b** Embryos treated with 6-OHDA. **c** Percentage of myelin formation along the tract (unpaired t test, p = 0.0037). N = 32 for the control, and N = 28 for the group treated with 6-OHDA

To deplete dopaminergic signaling, we used the specific toxin 6-hydroxydopamine (6-OHDA) to damage dopaminergic neurons [51, 52]. This treatment led to a reduction of dopaminergic neurons labeled by tyrosine hydroxylase (Additional file 7). With 6-OHDA treatment from 48 hpf (hours post fertilization) to 72 hpf, 39% embryos exhibited obvious myelin deficits. Compared to controls, embryos with drug treatment exhibited a lower percentage of myelin formation (Figure 4a–c).

**Fig. 5:**
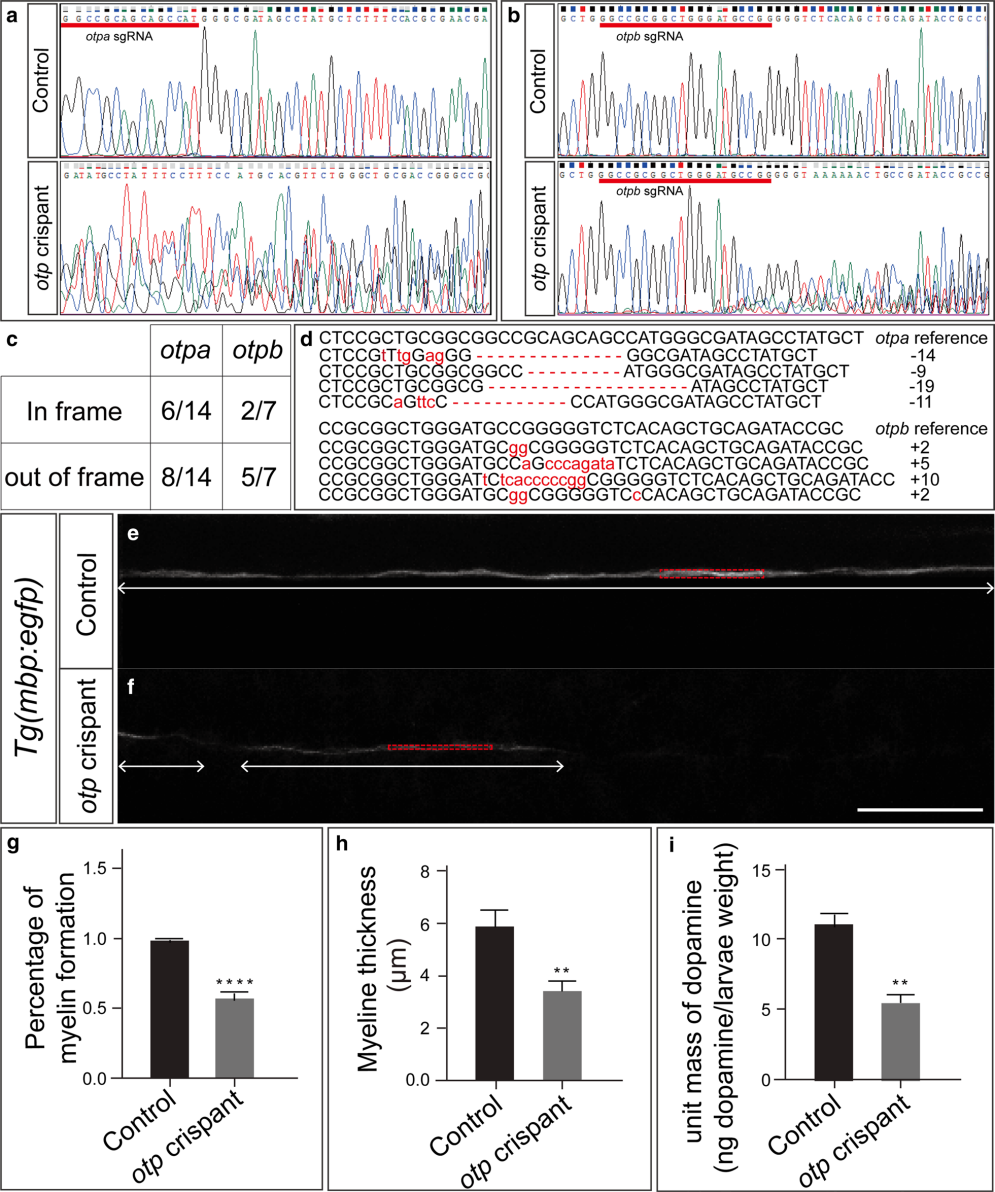
*otp* crispants have disrupted myelin. **a**, **b** An example of Sanger sequencing shows that *otpa* and *otpb* genes were disrupted after injection with CRISPR/Cas9. The sgRNA sequence is underlined in red. **c** Percentage of in-frame and out-of-frame mutations from *otpa* and *otpb* PCR amplicons (all PCR products have mutations). **d** Representative sequences from individually cloned PCR products. The sgRNA sequence is underlined in red. Insertions and deletions are shown as red letters and dashes, respectively. **e**, **f***Tg* (*mbp:egfp*) at 72hpf by confocal microscopy imaging. **e** control. **f***otp* crispant. **g h** Quantification of myelin deficits. N = 12 for the control group, and N = 15 for the *otp* crispant group. **g** Percentage of myelin formation along the tract (unpaired t test, p = 0.0001). Two-headed arrows (**e**, **f**) show the intact myelin. The lengths of intact myelin are added and the percentage is calculated by dividing by the length of the entire image window. The same threshold is set in each z projection for each embryo, and the length is calculated for visible segments. **h** Thickness of myelin sheaths (unpaired t test, p = 0.0045). The red box (**e**, **f**) is drawn around the visible myelin and the height of the box was used to calculate the thickness of the myelin sheaths. **i** ELISA shows that the dopamine levels (ng dopamine/larvae weight) are decreased in *otp* crispants (unpaired t test, p = 0.0079)

To confirm the specificity of the phenotype in embryos treated with 6-OHDA, we generated *orthopedia* (*otp* or *otpa/otpb*) crispants (F0 mosaic mutant animal) in which both *otpa* and *otpb* genes are disrupted (Figure 5a, b). *Otp* is a trancription factor important for dopaminergic neuron specification [53, 54]. As expected, dopamine levels were decreased in *otp* crispants as measured by ELISA (Figure 5i). Individual polymerase chain reaction (PCR) amplicons of *otpa* and *otpb* genes from injected embryos were cloned to confirm the extent of mutagenesis. Sequencing revealed that 14/14 *otpa* and 7/7 *otpb* PCR products exhibited mutations. Most amplicons were out of frame, which would lead to a stop and premature protein truncation (Figure 5c, d). Compared to controls, the *otp* crispants had a lower percentage of myelination (Figure 5e–g) and thinner myelin tracts (Figure 5e, f, h). Therefore, loss of dopaminergic signaling, either by 6-OHDA or CRISPR gene disruption, leads to myelin deficiency.

## Discussion

In this study, we used curated genetic-associated and transcriptome data to show that dopamine-related pathways are associated with MS. To our knowledge, this is the largest systematic analysis of MS-associated genes, which bridges the gap between dopaminergic signaling and demyelination. In addition, we confirmed the role of dopamine in myelination *in vivo* using zebrafish. Our *in vivo* analysis demonstrates that dopaminergic signaling is not just reflective of changes in myelination, but rather may play a causative role in MS.

Though it has been proposed that dopamine is associated with some symptoms in MS, for example, fatigue and defective immune response [55, 56], few studies, if any, have characterized the role of dopamine in myelin deficits in MS patients. In animal models, the dopamine receptor 2 (DAR2) responds during lithium restoration of myelin loss under stress [57]. Similarly, experimentally induced demyelination of the corpus callosum in mice can be ameliorated by dopamine D2 receptor antagonists [58]. The potential role of dopamine in myelin deficits observed in schizophrenia has attracted researchers’ attention. Myelin integrity was disturbed in multiple brain regions in patients with schizophrenia, as shown by diffusion tensor imaging (DTI). The antipsychotic clozapine, which at least partly binds to the dopamine receptor D2,can reverse this deficit in schizophrenia [7]. Interestingly, a strong inverse relationship between the dopamine D2/D3 receptor density and white matter integrity is found in health subjects, but this relationship appears to be disrupted in schizophrenia [8]. In healthy subjects, carriers of the CC genotype of the rs6277 polymorphism in the dopamine D2 receptor gene have elevated striatal dopamine turnover and higher myelin integrity in terms of fractional anisotropy [59]. The dopamine receptor agonist bromocriptine changes neural activity during attentional switching, also indicating a link between dopamine and white matter integrity [60]. The broad association between dopaminergic signaling and myelin in healthy subjects and patients with different neurological disorders suggests that the dopaminergic system could be a potential target for ameliorating myelination defects as well as improving cognition.

Demyelination, including that seen in MS, has deficits in the immune response and in the nervous system. However, its etiology has not been well characterized. On one hand, dopamine may play a complicated role in the immune response. The expression of dopamine receptor 5 (DAR5) in the peripheral blood mononuclear cells (PBMCs) is decreased in MS patients but progressively upregulated when treated with IFN-β [11, 12], though its exact role in the disease is not clear. In addition, DAR5 is also expressed in dendritic cells, which is critical for regulating the activity of different T cell targets [61]. These indicate the potential role of dopaminergic signaling in the innate and adaptive immune responses of MS. In our study, with both crosstalk analysis and transcriptome analysis, we found that the DS signaling pathway could interact or associate with the complement and coagulation cascade in both grey and white matter lesions, showing that dopamine may also function through the complement system, as a bridge between the innate and adaptive immune responses. We also found that the dopaminergic synapse pathway is associated with leukocyte transendothelial migration and cell adhesion molecules, suggesting that dopaminergic signaling may be involved in MS by regulating leukocyte adhesion and transmigration across the endothelium in the brain. One limitation of this study is that the association analysis only shows the expression regulatory relationships in gene sets without considering their direct interactions. Further studies are necessary to uncover the exact molecular pathways regulated by dopaminergic signaling in MS.

On the other hand, dopaminergic signaling is known for its role in neurodevelopment and neurodegeneration [62, 63]. For example, the dopamine receptor 3 (DAR3) may regulate myelin-like processes in culture [64] and stress-induced myelin loss can be ameliorated by the dopamine receptor 2 (DAR2) [57]. We found that the dopaminergic pathway may interact or associate with the glutamatergic synapse and neurotrophin signaling pathways, suggesting a complex role of dopamine in the nervous system. In addition, demyelination is always accompanied, at least partially, with remyelination [65]. It is possible that dopaminergic signaling may regulate both demyelination and remyelination in MS.

The pathways associated with the DS pathway in grey and white matters are quite different, which indicate distinct mechanisms for signaling in grey and white matter lesions. In white matter lesions, more pathways were associated with the DS signaling pathway, for example, Th17 cell differentiation and leukocyte transendothelial migration. This is consistent with previous findings that immune cells are mainly distributed in white matter rather than grey matter in MS [66, 67]. In grey matter, the DS signaling pathway is associated with pathways that fall into the categories of the nervous system and the immune system, indicating a complicated role for dopaminergic signaling in this disease. Dissecting the role of dopaminergic signaling in grey and white matter may offer novel therapeutic targets for MS. In future studies, it would be interesting to explore in greater detail the mechanisms by which dopaminergic signaling is involved in myelin pathogenesis.

## Conclusions

In this study, we did a literature search to systematically establish a complete set of genes which is associated with MS in humans and in which the dopaminergic synapse signaling pathway is enriched. Transcriptome analysis further confirms that the expression of multiple dopamine gene sets is affected in patients with MS. Moreover, we utilized zebrafish as a model to validate the effects of dopaminergic signaling on myelination, employing both pharmacological and genetic manipulation. This study may provide us with insight into the molecular mechanisms of myelin pathogenesis in the nervous system.

## Supplementary information

**Additional file 1.** Curated genes associated with MS.

**Additional file 2.** Enriched KEGG pathways in the curated MS genes.

**Additional file 3.** Protein-protein interactions between DS and oLC genes in Interactome.

**Additional file 4.** Directed Protein-protein interactions between the DS and oLC genes in Pathway Commons.

**Additional file 5.** The oLC pathways interacting with the DS pathway.

**Additional file 6.** Pathways correlated with the DS pathway in the datasets GSE26927 for grey matter and GSE38010 for white matter.

**Additional file 7.** A reduction of dopaminergic neurons by 6-OHDA.

## Data Availability

All data generated or analyzed during this study are included in this published article and its supplementary materials.

## Abbreviations

cAMP: Cyclic adenosine monophosphate
CNS: Central nervous system
CRISPR/Cas9: Clustered regularly interspaced short palindromic repeats/ CRISPR-associated protein 9
DA: Dopamine
DAR2: Dopamine receptor 2
DAR3: Dopamine receptor 3
DAR5: Dopamine receptor 5
DS: Dopaminergic synapse
DTI: Diffusion tensor imaging
EAE: Experimental autoimmune encephalomyelitis
*eGFP*: Enhanced green fluorescent protein
ELISA: Enzyme linked immunosorbent assay
GWAS: Genome-wide association study
IFN-β: Interferon β
KEGG: Kyoto Encyclopedia of Genes and Genomes
LCC: Largest connected component
*mbp*: Myelin basic protein
MS: Multiple sclerosis
oLC: Other literature-curated
PathNet: Pathways based on Network information
PBMCs: Peripheral blood mononuclear cells
PCR: Polymerase chain reaction
PD: Parkinson’s disease
PPIs: Protein-protein interactions
ROAST: Rotation gene set tests for complex microarray experiments
TCR: T-cell receptor
6-OHDA: 6-Hydroxydopamine
