# Supplementary figures and images for: Integrative systems and functional analyses reveal a role of dopaminergic signaling in myelin pathogenesis
Source: J Transl Med. 2020 Mar 2;18:109. doi: 10.1186/s12967-020-02276-1 (PMC7053059; doi:10.1186/s12967-020-02276-1)

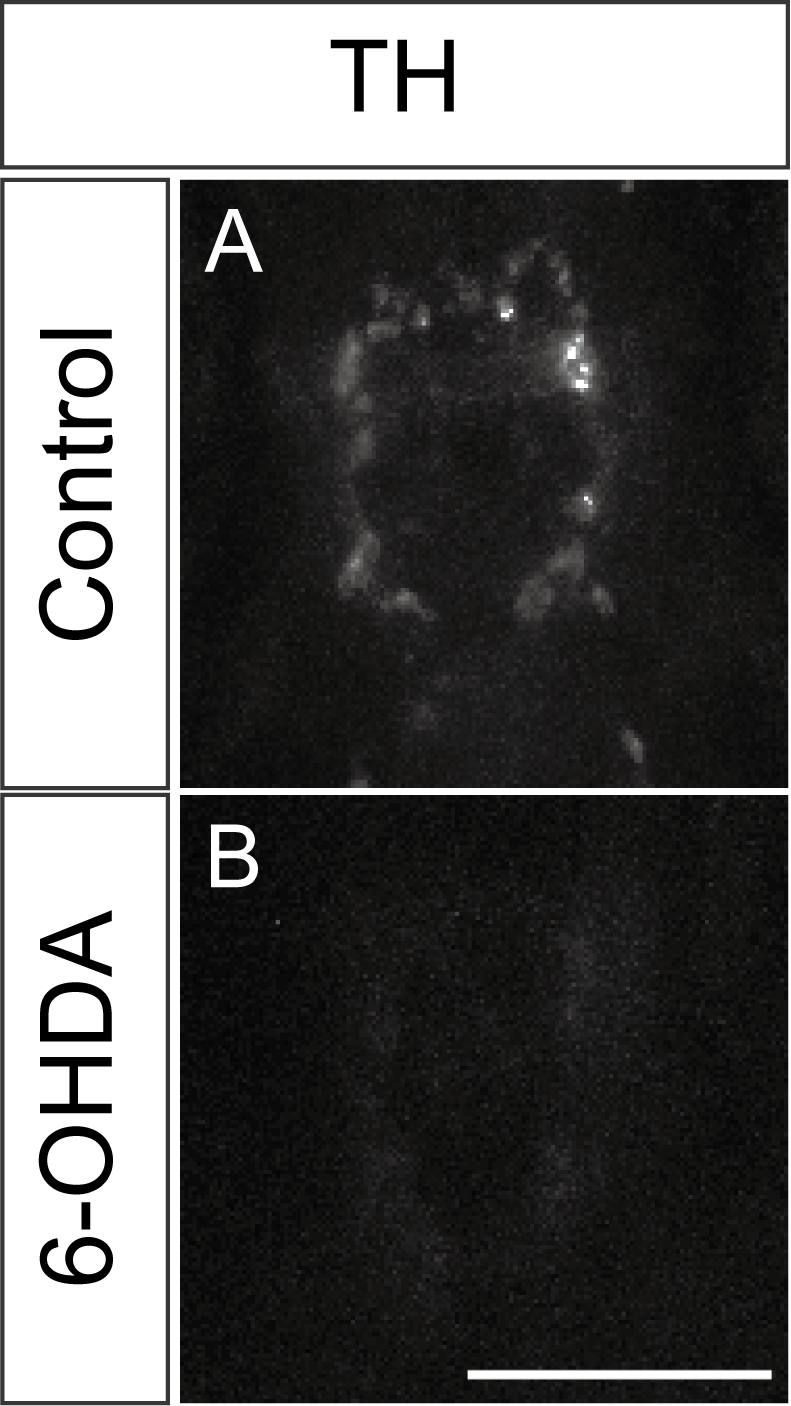

Supplement: Supplementary file 7 — Additional file 7. A reduction of dopaminergic neurons by 6-OHDA. [file 12967_2020_2276_MOESM7_ESM.tif]
